# Arsenic Toxicity on Metabolism and Autophagy in Adipose and Muscle Tissues

**DOI:** 10.3390/antiox11040689

**Published:** 2022-03-31

**Authors:** Seung-Hyun Ro, Jiyoung Bae, Yura Jang, Jacob F. Myers, Soonkyu Chung, Jiujiu Yu, Sathish Kumar Natarajan, Rodrigo Franco, Hyun-Seob Song

**Affiliations:** 1 Department of Biochemistry and the Redox Biology Center, University of Nebraska-Lincoln, Lincoln, NE 68588, USA; jbae42@wisc.edu (J.B.); abc211@hanmail.net (Y.J.); jacob.myers@students.jefferson.edu (J.F.M.); 2 Department of Cell and Regenerative Biology, University of Wisconsin School of Medicine and Public Health, Madison, WI 53705, USA; 3 Department of Neurology, Institute for Cell Engineering, The Johns Hopkins University School of Medicine, Baltimore, MD 21205, USA; 4 Laboratory of Immunology, Office of Biotechnology Products, Center for Drug Evaluation and Research, United States Food and Drug Administration, Silver Spring, MD 20993, USA; 5 Department of Microbiology and Immunology, Sidney Kimmel Medical College and Jefferson College of Life Sciences, MD-PhD Program, Thomas Jefferson University, Philadelphia, PA 19107, USA; 6 Department of Nutrition, University of Massachusetts, Amherst, MA 01003, USA; soonkyuchung@umass.edu; 7 Department of Nutrition and Health Sciences, University of Nebraska-Lincoln, Lincoln, NE 68583, USA; jyu18@unl.edu (J.Y.); snatarajan2@unl.edu (S.K.N.); 8 School of Veterinary Medicine and Biomedical Sciences and the Redox Biology Center, University of Nebraska-Lincoln, Lincoln, NE 68583, USA; rodrigo.franco@unl.edu; 9 Department of Biological Systems Engineering, University of Nebraska-Lincoln, Lincoln, NE 68583, USA; hsong5@unl.edu; 10 Department of Food Science and Technology, Nebraska Food for Health Center, University of Nebraska-Lincoln, Lincoln, NE 68588, USA

**Keywords:** arsenic, adipose, muscle, autophagy, oxidative stress, mitochondrial dysfunction, metabolism, metabolic syndrome, metabolic disease

## Abstract

Arsenic, a naturally occurring metalloid derived from the environment, has been studied worldwide for its causative effects in various cancers. However, the effects of arsenic toxicity on the development and progression of metabolic syndrome, including obesity and diabetes, has received less attention. Many studies suggest that metabolic dysfunction and autophagy dysregulation of adipose and muscle tissues are closely related to the development of metabolic disease. In the USA, arsenic contamination has been reported in some ground water, soil and grain samples in major agricultural regions, but the effects on adipose and muscle tissue metabolism and autophagy have not been investigated much. Here, we highlight arsenic toxicity according to the species, dose and exposure time and the effects on adipose and muscle tissue metabolism and autophagy. Historically, arsenic was used as both a poison and medicine, depending on the dose and treatment time. In the modern era, arsenic intoxication has significantly increased due to exposure from water, soil and food, which could be a contributing factor in the development and progression of metabolic disease. From this review, a better understanding of the pathogenic mechanisms by which arsenic alters metabolism and autophagy regulation could become a cornerstone leading to the development of therapeutic strategies against arsenic-induced toxicity and metabolic disease.

## 1. Introduction: Arsenic’s Impacts on Food and Human Health in the USA

US citizens consume significant amounts of agricultural products (e.g., rice grain from California or vegetables and fruits from Michigan) produced from the states with high exposure levels of arsenic (As) contamination [1,2,3]. This could correlate with the metabolic dysfunction caused by As accumulation in adipose (fat) and muscle tissues of the human body. For example, in Nebraska, the highest concentrations of As in groundwater, measured at over 20 μg/L (ppb), were found in the panhandle, southwestern region and Republican River valley [4], which belong to the Ogallala Aquifer in the Great Plains [5]. Nebraska is one of the largest beef-producing states, along with having high levels of corn and soybean production [6]. According to the United States Department of Agriculture (USDA) and Food and Drug Administration (FDA), As levels in beef, corn and soybean are quite low and no health concern has been found for most of Nebraska. Since cow livestock are fed with corn and soybeans and consume water from underground sources, there is still a possibility of a certain level of As accumulation in beef, corn and soybeans due to As contents in water and soil in highly As-contaminated areas. Indeed, multiple studies indicate that corn and soybeans are very susceptible to As uptake in highly As-contaminated areas [7,8]. Since beef mostly consists of fat and muscle tissues, measurements of As accumulation traveling from water and soil through to corn and soybeans, beef and finally to humans are increasingly sought, although there is no accurate database available due to various technical limitations in the measurement of live animals and humans in highly As-contaminated areas. These data relate to the USA as a whole, as the prevalence of As contamination in food production poses a significant concern due to the impacts on human health.

As, a group 1A carcinogen, is a worldwide public health concern; exposure causes lung, bladder and non-melanoma skin cancers in humans [9,10,11]. However, in contrast to extensive research on its carcinogenicity, considerably less studies are available on the impacts of As exposure on the onset and progression of other prevalent diseases, such as metabolic and degenerative diseases [12,13,14]. This is a critical knowledge gap, as several studies have suggested an association between chronic exposure of high-dose As and metabolic complications such as obesity and type 2 diabetes mellitus (T2DM), including from human studies in Michigan and population studies in high-arsenic areas (≥150 µg/L or ppm in drinking water) in Chile, Taiwan and Bangladesh [1,2,3,15,16,17,18].

A recent study estimated that about 2.1 million people in the USA alone are exposed to wells or underground water that is high in As [19]. Excess body weight and obesity combined make up the second leading cause of death in the USA [20]. The combined medical costs associated with the treatment of preventable obesity-associated diseases are estimated to increase by $48–$66 billion per year by 2030 in the USA [21,22]. It is important to note that obesity is often associated with dysplasia, lipodystrophy and malfunction of adipose tissue [17,23]. Disruption of glucose metabolism and accumulation of oxidative stress in the muscle [24,25] also significantly contribute to the development of metabolic diseases. Nevertheless, studies on the effects of As on adipose and muscle tissue metabolism, which are tightly linked to obesity and metabolic diseases, are limited. A better understanding of As’s instigating roles in metabolic dysfunction in adipose and muscle tissues could lead to innovative prevention and treatment strategies for people with obesity and metabolic diseases living in As-contaminated areas.

## 2. As Biochemistry and Metabolism: Biotransformation of As in the Body

As is a metalloid with an atomic number of 33 that is naturally found in the environment. As compounds can be classified into inorganic, organic and gas states. Inorganic As is generally considered more toxic than the organic form, while the gas form of As (Arsine) is the most toxic arsenical when acutely exposed in high concentrations [26]. As originates from the earth’s crust and is present at high levels in groundwater [27]. Humans can be exposed to As most significantly from contaminated groundwater by drinking, and indirectly at lower amounts from food (crops, fish, meat and dairy products prepared with As-contaminated water), industrial processes (alloying agents, metal adhesives, wood preservatives, pesticides, pharmaceuticals, etc.) or tobacco via cigarette smoking [28]. As can enter into the human body via ingestion through the gastrointestinal (GI) tract, inhalation through the lungs and dermal absorption. Absorbed inorganic As is metabolized in the human body with a half-life of two to four days [29], and approximately 60–80% of As is excreted in the urine through the kidneys within a few days of ingestion [30]. After absorption through the GI tract and lungs, As is taken up by red and white blood cells in the bloodstream first before reaching the liver [31].

Accumulated As in the body can generate ROS and epigenetic dysregulation, which promotes carcinogenesis. One study reported that the Rac family small GTPase 1 (Rac1) signaling pathway mediates As-induced generation of reactive oxygen species (ROS), which are well-known second messengers for cellular pathogenic effects by damaging protein, DNAs and organelles. Rac1 is an essential subunit of NADPH oxidase isoform Nox2, which is required for As-stimulated vessel remodeling during carcinogenesis [32,33]. Recent work found the links between As exposure to epigenetic dysregulation and progression of the pathology. During carcinogenesis, As exposure induces a loss of DNA methylation, which increases gene expression [34,35]. Arsenic-induced malignant transformations in human urothelial cells are associated with an aberrant DNA methylation increased by As exposure [36]. Therefore, the detoxification and secretion of absorbed As out of the body is the critical process in As biotransformation in order to remedy the toxicity.

The most efficient detoxification process of As in the human body is through methylation. As methylation is a biotransformation conserved from bacteria to humans to modulate As toxicity and carcinogenicity [37,38,39]. This process is catalyzed through S-adenosylmethionine (SAM)—dependent methyltrasferase (MT), which is encoded by the arsM gene [37,40]. Arsenate (As^V^) can be reduced to arsenite (As^III^) or methylated in the liver via enzymatic transfer of the methyl group from SAM to methyl arsonate (MMA^V^) and dimethyl arsenate (DMA^V^). However, methylation of inorganic As such as monomethylarsonous acid (MMA^III^) and dimethylarsinous acid (DMA^III^) would be toxic when interacting with cellular proteins and DNA [41,42]. When the methylating capacity of the liver is exceeded, As can be retained for two to four weeks or longer in soft tissues, including adipose and muscle tissues, as well as in keratin-rich tissues, including hair, nail and skin tissues [43,44].

As can be reduced by the antioxidant glutathione (GSH). As binding to the thiol (SH-) group is very strong and the reduction is initiated by shifts in the carbon atom bonded to the sulfhydryl group of the cysteinyl residue of GSH [45]. Dietary selenium (Se) compounds and α–tocopherol (Vitamin E) protect human cells and mice from As-induced mutagenesis and carcinogenesis [46].

Methylated trivalent inorganic As in the liver is released into the bloodstream and excreted in urine via transport through cell membranes. The groups of uptake and efflux channels facilitate As species transport, namely prokaryotic phosphate transporters (Pit and Pts); GlpF (glycerol facilitator protein) from *Escherichia coli*; AqpS (aquaporin) from *Sinorhizobium meliloti*); HxP (hexose permease) and Fps1p (yeast aquaglyceroporin) from *Saccharomyces cerevisiae*; and mouse aquaporin (AQP) 7, rat and human AQP9, glucose transporter (GLUT) 1 from mammals [47,48]. Additionally in mammals, GLUT2 and multidrug-resistance-associated protein (MRP) 2 play an important role in the efflux of dimethylarsenic metabolites (DMAs) [49,50]. These studies indicate that As transporters in each species have evolutionarily significant differences both in selectivity and transport rates of arsenicals.

Recently, there has been an emerging interest in the thiolation of As, which is a naturally occurring chemical reaction via adsorption on iron oxides and precipitation on iron sulfide minerals [51] and was found in geothermal waters in Yellowstone National Park [52] and in soil pore waters in rice fields [53,54]. The increasing synthesis and detection of thioarsenic species such as monomethylmonothioarsonous acid (MMMTA^III^), dimethylmonothioarsinous acid (DMMTA^III^), monomethylmonothioarsonic acid (MMMTA^V^), dimethylmonothioarsinic acid (DMMTA^V^) and dimethyldithioarsinic acid (DMDTA^V^) suggest the importance of these As metabolites in terms of cellular toxicity and therapeutic effects [55,56,57]. DMMTA^V^ is the most cytotoxic thioarsenic radical, similar to DMA^III^ in mammals [58]. Since the effects of thioarsenic exposure on human health are controversial, analytical and diagnostic methods for thioarsenicals are urgently needed.

Upon recent and chronic exposure to doses less than acute toxic concentrations (10 to 1000 ppb), 24 h urine collection is the most useful laboratory test. Other biomarkers such as measurement of the liver transaminase level or complete blood count (CBC) can be performed for clinical assessment of As exposure according to the guidelines from the Agency for Toxic Substances and Disease Registry (ATSDR, https://www.atsdr.cdc.gov/toxprofiledocs/index.html, reviewed and accessed on 22 February 2022.) under the Centers for Disease Control (CDC) in the USA. Patients with suspected acute As intoxication and poisoning generally require gut decontamination, hemodynamic stabilization and fluid and electrolyte replacement in an intensive care setting. As-chelating agents such as dimercaprol (2,3-dimercaptopropanol, also known as British anti-Lewisite or BAL), 2-3-dimercapto-1-propanesulfonate (DMPS) or meso 2,3-dimercaptosuccinic acid (DMSA) can be administered within hours of As absorption to prevent As-induced toxicity in human body [59,60,61].

**Figure 1:**
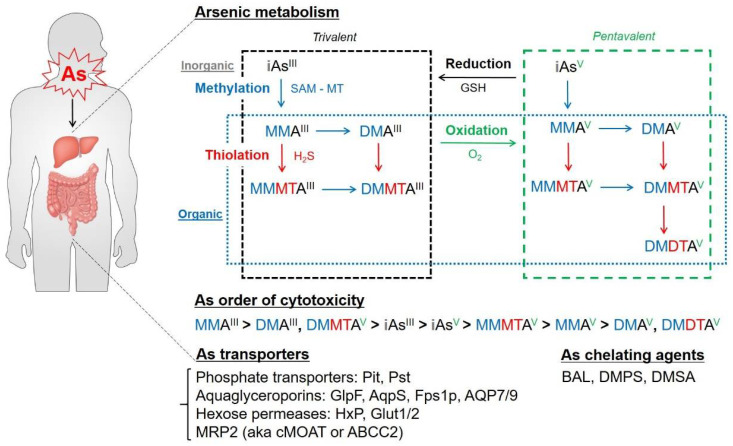
Biotransformation of arsenic in the human body. Once arsenic (As) is absorbed through the gastrointestinal tract and lungs from air, water, food and the environment, various As species are generated by biotransformations, such as methylation and thiolation. As converts its oxidation states between pentavalent (+5) and trivalent (+3) via the reduction and oxidation processes in the human body. As is transported via aquaglyceroporin channels on cellular membranes and mobilized through the bloodstream. Toxic As species are detoxified in the liver and secreted through urine. However, As species over the detoxification threshold of the liver can be further mobilized and accumulated in the human body for several months or even longer. As-chelating agents such as BAL, DMPS and DMSA are frequently used for clinical and therapeutic purposes against acute As exposure and toxicity. Note: iAs^III^, inorganic trivalent arsenic; iAs^V^, inorganic pentavalent arsenic; MMA^III^, monomethylarsonous acid; DMA^III^, dimethylarsinous acid; MMA^V^, methyl arsonate; DMA^V^, dimethyl arsenate; MMMTA^III^, monomethylmonothioarsonous acid; DMMTA^III^, dimethylmonothioarsinous acid; MMMTA^V^, monomethylmonothioarsonic acid; DMMTA^V^, dimethylmonothioarsinic acid; DMDTA^V^, dimethyldithioarsinic acid; SAM—MT, S-adenosylmethionine-dependent methyltrasferase; GSH, glutathione; Pit, low-affinity, high-capacity phosphate transporter; Pst, high-affinity, low-capacity phosphate transporter; GlpF, glycerol facilitator protein; AqpS, aquaporin S, Fps1p, yeast aquaglyceroporin; AQP7/9, aquaporin 7/9; HxP, hexose permease, Glut1/2, glucose transporter 1/2, MRP2, multidrug-resistance-associated protein 2; cMOAT, canalicular-multispecific organic anion transporter 1; ABCC2, ATP-binding cassette sub-family C member 2; BAL, 2,3-dimercaptopropanol; DMPS, 2-3-dimercapto-1-propanesulfonate; DMSA, meso 2,3-dimercaptosuccinic acid. The order of As species in terms of their cytotoxicity levels was organized by referring to the previous publications (see main text). The blue, red, black and green arrows indicate methylation, thiolation, reduction and oxidation processes, respectively.

We briefly reviewed the As biotransformation and clinical treatment methods in humans. Since As-associated diseases such as cancer and metabolic diseases may have a long latency period, many As-exposed patients remain asymptomatic for years. Therefore, the understanding of As metabolism and the clinical manifestation based on the As exposure route, dose, chemical form or species, frequency, intensity, duration and elapsed time from As-exposed individuals or patients would be critical for prevention and treatment of As-associated toxicity and diseases (Figure 1).

## 3. Effects of as on Lipid Metabolism and Adipocyte Function

As accumulates in various tissues in the body, including liver, lung, brain, heart, kidney, eye and spleen tissues [62]. Chronic exposure to high levels of As has been associated with metabolic diseases such as cardiovascular diseases, hepatotoxicity, diabetes, obesity and cancers [17,63]. Lipid metabolism plays an important role in chronic diseases and metabolic syndrome [64,65]. A 15-year birth cohort study showed that early-life As exposure caused higher atherogenic low-density lipoprotein (LDL) cholesterol levels in Taiwanese adolescents [66]. As induced dyslipidemia and hypercholesterolemia in male albino rats after 12 weeks of 150 mg/L (ppm) As exposure in drinking water [67], but this study used a very high dose and would be expected to be associated with acute liver toxicity. This would not be relevant to chronic effects in humans at environmental exposure levels—even at relatively high exposure levels. As inhibited high-density lipoprotein (HDL) cholesterol and increased plasma-free fatty acids. The expression levels of cholesterol metabolism genes, including sterol regulatory element-binding protein 1 (Srebp1c), 3-hydroxy-3-methylglutaryl-CoA reductase (Hmgcr) and cytochrome P450 family 7 subfamily A member 1 (Cyp7a1), were significantly decreased in the liver samples of mice exposed to 0.25 ppm As in drinking water for two weeks [68]. In addition, serum cholesterol and lipid levels were significantly increased in mice treated with 1 ppm As for two weeks [68]. Furthermore, lipid peroxidation was increased in the brains of rats exposed to 25 ppm of As compared to the control group, suggesting that As induced ROS production in the rat brain [69]. High-dose exposure of As also disrupts lipid metabolism in the adult zebrafish liver, as revealed by decreased mRNA expression of carnitine O-octanoyl transferase (Crot) and 3-hydroxy-e-methylglutaryl-CoA synthase 1 (Hmgcs1) and increased mRNA expression of fatty acid binding protein-3 (FABP3) [70]. As exposure promotes atherosclerosis in the aorta of rats by decreasing the HDL cholesterol/LDL cholesterol ratio [71]. The level of an indicator of lipid peroxidation, malondialdehyde (MDA), was increased in the serum of rats exposed to 3 ppm As in drinking water throughout development until the adult stage [72]. Additionally, serum adiponectin, triglyceride and HDL cholesterol levels were significantly decreased in mice after 18 weeks of exposure to 50 ppm As in their drinking water [73]. Collectively, these studies demonstrate that As can affect lipid metabolism as well as cholesterol metabolism at acute high-dose exposure levels.

**Figure 2:**
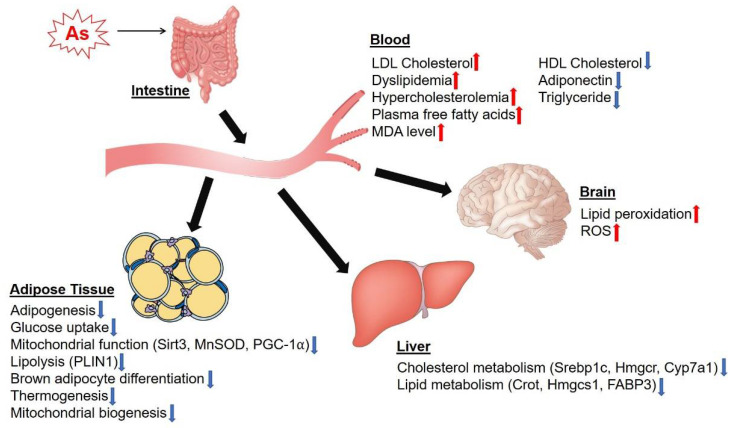
The effects of As on cholesterol and lipid metabolism in various tissues. When high-dose As uptake occurs in the intestines, As is delivered to metabolic tissues such as liver and adipose tissues. The accumulation of As in adipose tissues resulted in the inhibition of adipogenesis, mitochondrial function and thermogenic function. The dysregulation of lipid metabolism caused by As in adipose tissue affects lipid and cholesterol levels in the blood, and eventually alters lipid and glucose metabolism in other metabolic tissues such as the liver and muscles. Note: LDL, low-density lipoprotein; HDL, high-density lipoprotein; MDA, malondialdehyde; ROS, reactive oxygen species; Sirt3, sirtuin 3; MnSOD, manganese superoxide dismutase; PGC-1⍺, peroxisome-proliferator-activated receptor-gamma coactivator 1⍺; PLIN1, perilipin; Srebp1c, sterol regulatory element-binding protein 1; Hmgcr, 3-hydroxy-3-methylglutaryl-CoA reductase; Cyp7a1, cytochrome P450 family 7 subfamily A member 1; Crot, carnitine O-octanoyltransferase; Hmgcs1, 3-hydroxy-e-methylglutaryl-CoA synthases 1; FABP3, fatty acid binding protein-3. The red arrow indicates an increased effect, while the blue arrow indicates a decreased effect.

As plays a role in adipocyte differentiation and adipose tissue dysfunction and lipodystrophy [74]. As inhibited adipogenesis and glucose uptake in various adipocyte cell types, including 3T3-L1 preadipocytes, mouse-adipose-derived stromal vascular fraction cells and human adipose tissue-derived stem cells [75]. The level of mitochondrial oxidative stress response protein, sirtuin (Sirt) 3, was decreased in As-treated 3T3-L1 adipocytes and C2C12 myotubes, leading to mitochondrial damage [76]. Moreover, the expression levels of Sirt3 targets, including manganese superoxide dismutase (MnSOD) and peroxisome-proliferator-activated receptor-gamma coactivator (PGC)-1α, were decreased in As-treated 3T3-L1 and C2C12 cells, indicating that As regulates ROS and its related metabolism in adipocytes [76]. As also inhibits the differentiation of C3H10T1/2 mouse adipose stem cells, resulting in inhibition of lipid accumulation [77]. Chronic exposure to As reduces the adipose tissue expression of perilipin (PLIN1), which regulates lipid storage and lipolysis [78]. Moreover, As also regulated browning and brown adipocyte function. As reduced adipocyte differentiation, lipogenesis, thermogenesis and mitochondrial biogenesis in murine brown adipocytes and brown adipose tissue (BAT) [79,80]. Mice exposed to 5 ppm As in their drinking water showed cold intolerance, as revealed by the whitening of BAT [80]. In addition, the expression levels of genes that regulate thermogenesis, mitochondrial function, adipogenesis and lipolysis were decreased in BAT samples from mice exposed to 5 ppm As for 17 weeks in their drinking water [80]. Taken together, these results provide evidence that As inhibits adipogenesis, mitochondrial function and BAT thermogenic functions (Figure 2).

## 4. Effects of As on Glucose Metabolism, Mitochondrial Energy Metabolism and Muscle Function

Most of the As studies in muscle focused on fish meat (e.g., tuna, tilapia, killifish) as a food source [81,82,83,84]. Arsenite-induced oxidative stress induced autophagy as a defense mechanism in chicken muscle [85]. In a mouse study, up to 0.5 ppm of As trioxide (As_2_O_3_) caused myoatrophic effects with decreased muscle mass and endurance [86]. In a recent human study, muscular atrophy was observed as a side effect in acute promyelocytic leukemia patients treated with As trioxide [87]. While this study used doses of As that exceeded those found in environmental exposure, this is important to acknowledge because it shows the connection between As and muscle atrophy in a human model. Arsenic exposure increases the reductions in skeletal muscle mass [88] and increases the genetic risk factors in muscle atrophy and cardiovascular disease [89] in humans. A chronic low-dose As treatment (0.25–1 µM of As trioxide) causes C2C12 myotube atrophy by inhibiting the AKT pathway [90]. Since humans consume low doses of As chronically through As-contaminated food sources such as fish and chicken, a quantitative analysis of As accumulation in skeletal muscle in humans and how those chronically accumulated As exposures can affect the glucose metabolism in muscle tissue is required.

As exposure inhibits myogenesis by inhibiting the β-catenin pathway [91]. As interferes with gluconeogenesis in muscle by inhibiting glucose transporters and suppressing regulatory genes in glucose metabolism [92]. Thus, As can substantially interfere with glycolytic and mitochondrial energy generation. However, the exact mechanism explaining the function of As as an antagonist or synergist in glucose homeostasis in muscle tissue remains to be understood. Biologically relevant oxidation states of As, namely trivalent (As^III^) and pentavalent (As^V^) forms, are interconvertible and detoxified through methylation in mammals [93,94]. Both oxidation states may have different chemistries and interactions with glucose metabolism and energy metabolism pathways in adipose and muscle tissues, although the relevance of As metabolism in adipose and muscle tissues with human health outcomes requires further study. While As poisoning and exposure may seem to be a rare and relatively insignificant issue, in reality hundreds of millions of people are chronically exposed to elevated levels of As [95] through contaminated drinking water [96], crops and livestock [97]. This chronic exposure can cause a substantial disease burden in exposed populations [98], with a prevalence of As-mediated disease directly relating to the level of exposure [95]. It is worthy of note that there is a threshold for As-related diseases. One study suggests that the low exposure range of iAs over 150 ppb would increase the risk of lung and bladder cancers [99]. Large epidemiology studies in Japan and the USA show no increased risk of cardiovascular disease with even lower exposure levels of iAs under 100 ppb related to amount of rice consumed, the grain with the highest As levels [100]. There are no threshold data available for human studies in metabolic disease, such as obesity and diabetes, meaning the levels of chronic iAs contamination required to influence the emergence of these diseases in a population could be much lower than for cancer.

In addition to impacting a host of biological systems, an important aspect of As-mediated disease is its ability to mimic the symptoms of diabetes through the interactions with glucose metabolism [101]. In vitro studies have demonstrated that treatment with sub-toxic levels (1 mM, Table 1) of As^III^, in both its inorganic and metabolized mono- and di-methyl species, significantly reduced insulin mediated glucose uptake in 3T3-L1 adipocytes by interfering with the localization of insulin-responsive glucose transporter 4 (Glut 4) to the cell membrane [102]. Additional in vitro studies found that longer term exposure of 3T3-L1 adipocytes to lower levels of inorganic As^III^ (iAs^III^) significantly reduced insulin-mediated glucose uptake in levels as low as 0.5 µM [76]. These findings are recapitulated in in vivo mouse models [101], and additional studies indicate that As^III^ may inhibit the secretion of insulin from pancreatic cells [103]. A proposed mechanism for the reduced insulin secretion is via the inhibition of mitochondrial metabolism in pancreatic β-cells [104]. A prospective cohort in humans exposed to very high As levels indicated that the diabetes risk increased by 3% for every 1 ppb of As present in drinking water [105]. In 2001, the USA Environmental Protection Agency (EPA) revised the acceptable level of As in drinking water down from 50 ppb to 10 ppb. As is also capable of directly impairing glucose metabolism. Some evidence suggests that inorganic As^V^ (iAs^V^) can directly interact with glycolytic pathways via substitution with phosphate [106], although this study was conducted using only enzymes and substrates and required iAs^V^ to be at twice the concentration of phosphate to observe any effect, while arsenate levels would never be that high in vivo as that would be lethal, so the biological significance of As on glycolysis is not clear.

Clearer evidence exists relating As to mitochondrial dysfunction. When isolated rat mitochondria were incubated with 10 to 100 µM of iAs^III^, mitochondrial respiratory complexes I and II were inhibited, leading to excess ROS production, membrane potential collapse and release of cytochrome c [107]. These findings were reproduced with in vivo rat studies of chronic As exposure, inhibiting the activity of complexes I, II and IV [108,109], while the mRNA levels of mitochondrial genes in complexes I and IV were downregulated following chronic As exposure [109]. In vivo studies in rats exposed to levels of As commonly found in contaminated drinking water for 30 days found significantly reduced activity levels of several key enzymes of the citric acid cycle, including isocitrate dehydrogenase (ICDH) and succinate dehydrogenase (SDH) [108]. The inhibition of mitochondrial complexes following As exposure has important implications, as the ROS produced by As-exposed mitochondria has been shown to be the major route of As-mediated mutagenesis at cytotoxic concentration (0.25 μg/mL, Table 1) [110]. Thus, the generation of mitochondrial ROS by As can be linked to the role of As as a carcinogen [111]. Most of the current cell culture and animal experiments used lethal concentrations (Table 1 shows the doses and durations of As administration). Nearly all of the mitochondrial effects of As are at acutely toxic, poisonous and lethal concentrations. While these studies were conducted at high As concentrations, they provide valuable insight into the basic mechanisms of how chronic lower level As exposure might cause or worsen metabolic diseases.

As exposure also poses important implications for muscle function. Mice exposed to 100 ppb of iAs^III^ for five weeks showed decreased muscle function, damage to muscle progenitor cells and a significant increase in non-phosphorylating LEAK respiration [112]. This indicates that the reduced muscle performance stemming from As may be a result of mitochondrial abnormalities. As is further implicated in muscle damage by inhibiting muscle repair, increasing nuclear factor kappa light-chain enhancer of activated B cells (NF- κB) inflammation signaling and resulting in increased healing time and fibrosis [113]. In addition to direct damage to muscle by As, the tendency for As to mimic diabetes can also impact muscle tissue [114]. Muscle atrophy can stem directly from the neuropathy caused by diabetes [115], and a reduction in isometric muscle performance was observed in diabetic patients during hyperglycemia [116]. Diabetes is also associated with an increase in intramuscular fat storage, which is in turn associated with reduced muscle strength [117].

**Figure 3:**
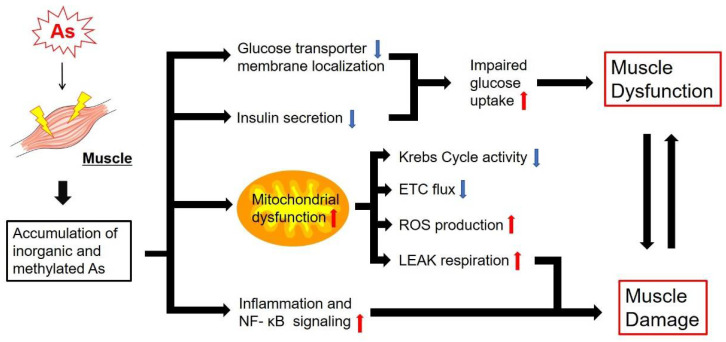
The effects of As on glucose metabolism and mitochondrial function in muscle tissue. When inorganic and methylated (organic) As are generated upon As absorption by muscle tissue, accumulated As species alter glucose metabolism and cause mitochondrial dysfunction. Accumulated As species also cause inflammation via NF-κB signaling in muscle tissue. As toxicity triggering muscle dysfunction and damage worsens metabolic-disease-associated pathologies. Note: ETC, electron transport chain; ROS, reactive oxygen species. The red arrow indicates an increased effect, while the blue arrow indicates a decreased effect.

In summary, As is associated with significant alterations to glucose metabolism and mitochondrial function with clear ability to deleteriously impact skeletal muscle function. A better understanding of how environmental As can impact muscle metabolism and function would be essential for developing a therapeutic method for humans suffering from As-mediated metabolic diseases (Figure 3).

## 5. Effects of as on Autophagy in Adipose and Muscle Tissues

Several studies indicated that As is known to have detrimental effects on adipose and muscle tissues of fish, animals, livestock, experimental rodents and humans, as discussed above. In the case of adipose tissue, many research groups have already evaluated the effects of As using various adipocytes such as 3T3-L1, 3T3-F442A, C3H 10T1/2, HIB1B and so on [77,79,118,119,120,121] (Table 1). Additionally, several animal experiments were also performed with As exposure [17,79,118] (Table 1). Most of these studies showed the inhibitory effects of As administration on the growth and differentiation of the adipocytes. Although many genes and signaling pathways have been suggested as possible causes, including peroxisome-proliferator-activated receptor gamma (PPARγ), CCAAT/enhancer-binding protein-alpha (C/EBPα), cyclin-dependent kinase inhibitor 1A (p21), protein kinase B (Akt/PKB), sirtuin 3 (SIRT3)-forkhead box O3 (FOXO3a), mitogen-activated protein kinase (MAPK) cascade and C/EBP homologous protein (CHOP10)-G protein coupled receptor (GPCR) pathways, the role of As in adipose tissue remains unclear [17,74]. Muscle is also known to be another target tissue of As exposure, although this is much less studied even than adipose tissue. One study showed that the levels of glucose transporters 1 and 4 (GLUT1/4) and activation of inositol phospholipid 3-kinase (PI3K) increased with 0.5 mM As treatment in L6 rat skeletal muscle cells [118,122]. In our previous study, we observed the accumulation of As in a dose-dependent manner in mouse adipose tissue [79]. However, in the case of muscle tissue, another study reported that there was no As accumulation in muscle tissue when mice were exposed to As [17,112]. Although As did not accumulate in the muscle tissue of mice, they found impaired muscle function and increased mitochondrial oxygen consumption through the isolated primary muscle cells from mice after As exposure of 100 μg/L for 5 weeks [112]. Interestingly, in our previous studies, we also measured the oxygen consumption rate in response to high-dose As treatment in adipocytes (10 μM) and in adipose tissue of mice (10 mg/kg/d). However, the oxygen consumption was significantly decreased in the adipocytes when the treated As concentration was increased, and there was no difference in mice when compared with the control group [79]. The working mechanisms of As in adipose and muscle tissues are unique, presumably due to sensitivity to As and differences in cellular adaptivity regarding As accumulation. Muscle weakness is a major factor contributing to decreased functional mobility and is a strong predictor of mortality. Muscle weakness and wasting are also some of the most common pathologic clinical symptoms of environmental exposure to As [86,88]. However, the mechanisms and molecular pathogenesis in the etiology of As-induced muscle morbidity are relatively unknown. In addition, although the contribution of As exposure to stem cell function is important for development and has been increasingly investigated, there is an unmet need to know whether and how environmental toxicants such as As affect adult stem cell behavior. It is important to resolve how stem cell vulnerability to environmental contaminants affects the ability of otherwise healthy tissues to respond to acute injury. An enhanced understanding of the mechanisms underlying the clinical symptoms of skeletal muscle dysfunction induced by As is crucial for the design of strategies and policies to prevent or reduce muscle injury. 

Autophagy, a topic of the 2016 Nobel Prize in Physiology or Medicine [123], is one of the major cellular degradation systems as a catabolic process of lysosomes for cellular life preservation [124,125,126]. Degenerated proteins, organelles and cytoplasmic wastes are removed by the autophagy system [127]. The objects for removal are isolated in the vesicles called autophagosomes, which form double-membraned structures in the cells. After the vesicle is combined with the lysosomes, autophagolysosomes are formed and then degraded by lysosomal enzymes [128,129]. The degraded materials are used to make energy for cell survival or to generate new organelles [130]. Thus, autophagy is a substantial intracellular recycling system that is used to protect cells from the accumulated toxic proteins and to maintain intracellular homeostasis [124]. Autophagy defects have been implicated in various metabolic diseases and cancer [131,132]. Autophagy is also necessary for proper differentiation of adipose tissue [133], and obese patients showed an abnormally high level of autophagy gene expression in white adipose tissue analyzed via bariatric surgery [134]. This could be a compensatory effect from the deficiency in autophagy activity and the elevation of the apoptosis pathway in obese patients [135,136]. Autophagy promotes muscle wasting and sarcopenia, a loss of muscle mass upon aging, because of protein breakdown. However, autophagy actually maintains muscle mass and protects against age-related muscle degeneration and dysfunction [137]. Increased basal autophagy protects against sarcopenia by degrading misfolded proteins and organelles. Although there are strong correlations between autophagy and metabolism, there is a knowledge gap on how autophagy regulates lipid and glucose metabolism in adipose and muscle tissues.

It is already well known that autophagy is closely connected in adipogenesis through studies in autophagy-deficient cell lines and mouse models, such as Atg5 and Atg7 knockouts [138]. Although impaired autophagy function from the embryo stage was fatal in mice after birth [139], in another sense autophagy-related gene knockout mice showed antiobesity and antidiabetic phenotypes in adipocytes [138]. Ironically, this depends on the conditions, but autophagy shows ambivalence. Although there is a lack of definitive research supporting the causal relationship between autophagy and As in adipose and muscle tissues, it has been shown that there is a close correlation between As toxicity and autophagy function [79,85,140,141]. In one research group, the upregulated autophagy-related proteins such as microtubule-associated protein 1 light chain 3 (LC3)-II and sequestosome 1 (p62) were observed in mouse skeletal muscle upon As exposure (4 μM or 4 mg/L in drinking water for 12 weeks) [141]. In another study, the accumulated As (Table 1) in chicken skeletal muscle induced intracellular oxidative damage and was linked to the autophagy activation by PI3K–AKT–mechanistic target of rapamycin kinase (mTOR) pathway inhibition and mitochondrial dysfunction [85,140]. In our previous studies, we also showed that As exposure induced oxidative stress and suppressed adipogenesis and mitochondrial biogenesis, respiration and thermogenesis via autophagy inhibition in adipocyte cell culture and adipose tissue of mice. The cause of the autophagy inhibition by As was associated with the suppressed sestrin-2 (SESN2) and unc-51-like autophagy-activating kinase 1 (ULK1) in BAT of mice [79].

The acute exposure to As trioxide (50 μM for 24 h) induced autophagy as a result of blocking the apoptosis pathway in H9c2 rat cardiomyoblastoma cells [142]. Acute autophagy induction in As exposure in mammalian cells is known to be significantly induced by ROS [143]. Contrary to studies in cardiomyocytes, multiple studies have demonstrated autophagy inhibition by As in NIH3T3 fibroblasts. One study reported that short-term treatment of 1 μM As for 4 h began to block autophagy flux by activating the Nrf2 pathway in a p62-dependent manner in NIH3T3 fibroblasts [144]. Another study observed that 2 μM As (III) treatment up to 48 h caused autophagy dysfunction by inhibiting SNARE complex formation in NIH3T3 fibroblasts [145].

**Figure 4:**
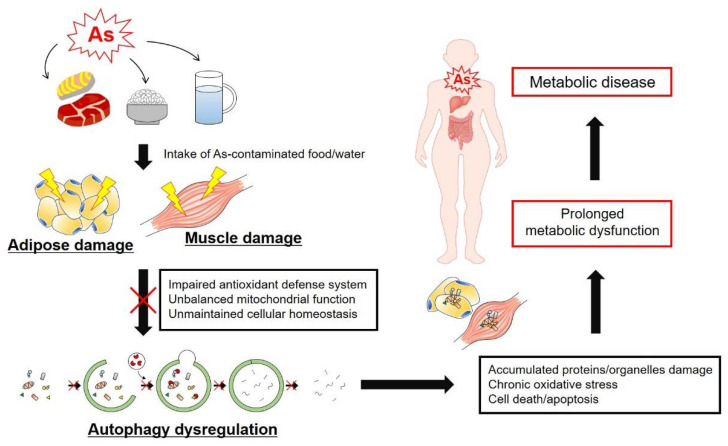
Autophagy dysregulation caused by chronic As exposure in adipose and muscle tissues contributes to the development of metabolic disease. Intake of As-contaminated food and water resulted in the long-term accumulation of As in adipose and muscle tissues. As toxicity increased the metabolic dysfunction, which can lead to metabolic disease in humans.

We have summarized the effects of As exposure on autophagy induction and function in adipose and muscle tissues. Although autophagy activation or inhibition by As is dependent on the As dose and length of treatment, cell type and mouse species, chronic As accumulation beyond the physiological threshold leads to autophagy dysregulation accompanied by an impaired antioxidant defense system, damaged intracellular organelles with metabolic dysfunction and unbalanced metabolic homeostasis in adipose and muscle tissues. Thus, autophagy dysregulation caused by chronic As exposure in adipose and muscle tissues contributes to the development of metabolic disease in humans (Figure 4).

## 6. Conclusion: Perspective on the Connection between As Exposure and Metabolic Disease

Historically, iAs, due to its odorless and tasteless properties, was used to kill many kings and emperors, and is known as the “poison of kings and the king of poisons” [146]. In BC 55, the Roman emperor Nero poisoned his step-brother Tiberius Britannicus with As to secure his position as Roman emperor. In contrast, in ancient Greece and China, Hippocrates and Chinese physicians used As as a treatment for certain types of disease, such as ulcers and abscesses, as well as for remedies against As-induced poisoning as a “poison against a poison” medicine [147]. Since then, As has been used as a chemotherapeutic method to cure syphilis, parasites and leukemia [148,149]. As has been used as a poison as well as a medicine, highlighting that the right amount of As in treatment could be beneficial for human health. In that sense, we could not exclude the possibility that chronic low-dose (less than 10 ppb) As exposure in adipose and muscle tissues would be beneficial as a hormesis effect for inducing the oxidative defense mechanism or inhibiting apoptosis differently from the harmful effects of high-dose As exposure in the liver, urinary tract, lungs and skin. In this review, we have summarized the effects of As exposure on metabolism and autophagy in adipose and muscle tissues, which are major target tissues in metabolic syndrome. Although the effects of acute and chronic As exposure have identified action mechanisms in adipose and muscle tissues, the effects of indirect As exposure from food or directly from the environment remain controversial and require further research in the USA. In conclusion, studies on the effects of As in metabolic function and in autophagy activity in adipose and muscle tissues could be beneficial for the group of people who are suffering from metabolic syndrome and diseases in highly As-contaminated areas worldwide.

**Table 1:** Summary of the maximum dose and duration of As treated or administered in the research papers that are referenced in this review.

Samples		Dose and Duration of As	Reference
Cell culture	3T3-L1 preadipocytes, mouse-adipose-derived stromal vascular fraction cells and human adipose tissue-derived stem cells	5 μM (sodium arsenite), 1 μM (trivalent monomethylated arsenic), or 2 μM (trivalent dimethylated arsenic) in medium, 2 days with DMI (differentiation media)	[75]
	3T3-L1 adipocytes	1 mM, sodium arsenite (NaAsO_2_), methylarsine oxide and iododimethylarsine in medium, until full differentiation for several days	[102]
	3T3-L1	3 μM, arsenic trioxide (As_2_O_3_) in medium, during differentiation for 24 h	[119]
	3T3-F442A	0.5 μM/L, arsenic trioxide in medium, 3 days	[120]
	Adipose-tissue-derived primary human mesenchymal stem cells (hMSCs)	1 μM, sodium arsenite in medium, 72 h	[78]
	C2C12 myoblast cells and 3T3-L1	2 μM, sodium arsenite in medium, 8 weeks	[76]
	C2C12 myotubules	1 μM, arsenic trioxide in medium, 48 h	[90]
	C3H 10T1/2 adipocytes	6 μM, sodium arsenite in medium, 2 months	[77,121]
	EJ-1, human bladder cancer cells	Up to 1 mM (iAS^III^, iAS^V^, MMA^V^, MMMTA^V^, DMA^V^, DMA^III^,DMMTA^V^ or DMDTA^V^) in medium, 24 h	[58]
	H9c2 rat cardiomyoblastoma cells	50 μM, arsenic trioxide in medium, 24 h	[142]
	HaCaT human keratinocytes	25 μM, sodium arsenite in medium, 24 h	[35]
	HEK293, NIH3T3, BEAS-2B	1 μM, sodium arsenite in medium, 24 h	[144]
	HIB1B adipocytes	10 μM, sodium arsenite in medium, 6 days	[79]
	Human–hamster hybrid A_L_ cells	0.25 μg/mL, sodium arsenite in medium, 60 days	[110]
	INS-1 832/13 beta cells	2 μM (sodium arsenite, dimethyl arsenite), 0.5 μM (mono-methyl arsenite) in medium, 24 h	[104]
	L6 rat skeletal muscle cells	0.5 mM, sodium arsenite in medium, 30 min	[123]
	NIH3T3, HeLa cells	2 μM, sodium arsenite in medium, 48 h	[145]
	P19 mouse embryonic stem cells	1 μM, sodium arsenite in medium, 5 days until full differentiation	[91]
	PAEC, porcine aortic endothelial cells	5 μM, sodium arsenite in medium, 2 h	[32]
	Pancreatic eyelets (ex vivo) of B6 mice	0.1 μM (methylarsonite, dimethylarsinite) in medium, 48 h	[103]
	Primary SECs, Liver sinusoidal endothelial cells	5 μM, sodium arsenite in medium, 8 h	[33]
	UROtsa, Non-tumorigenic human urothelial cell lines	1 μM, sodium arsenite in medium, 12 weeks 50 nM, MMA (III) in medium, 24 weeks	[36]
Animals	Chicken skeletal muscle (in vivo)	2.5 mg/kg/day, until reach 30 mg/kg, H_3_AsO_3_ in diet	[85]
	Chickens	2.5 mg/kg, As_2_O_3_ in diet, 12 weeks	[140]
	Hamster LD_50_	112 μmol/kg and 29.3 μmol/kg (MMA^III^) in diet	[42]
	Mice (C57BL/6 aka B6, male and female)	10 mg/kg/d, sodium arsenite in gavage, 9 days	[79]
	Mice (B6, male)	45 ppm, sodium arsenite in drinking water, 48 weeks	[34]
	Mice (B6, male)	50 ppm, sodium arsenite in drinking water, 18 weeks	[73]
	Mice (B6, male)	50 ppm, sodium arsenate in drinking water, 8 weeks	[101]
	Mice (B6, female)	20 ppm, sodium arsenite and sodium arsenate dibasic heptahydrate (1:1 ratio) in drinking water, 17 weeks	[80]
	Mice (C57BL/6J, male) eye	250 ppm for 1 month or 50 ppm for 6 months (sodium arsenite) in drinking water	[62]
	Mice (B6) skeletal muscle	4 μM or 4 mg/L, arsenic trioxide in drinking water, 12 weeks	[141]
	Mice (C57BL/6Tac)	100 μg/L, sodium arsenite in drinking water, 5 weeks	[78]
	Mice (C57BL/6Tac)	100 μg/L, trivalent arsenite (AsIII) in drinking water, 5 weeks	[112]
	Mice (C57BL/6NTac male)	100 μg/L, sodium metaarsenite (NaAsO_2_) in drinking water, 5 weeks	[113]
	Mice (C57BL/6Ai p47^phox^ knockout)	250 ppb, sodium arsenite in drinking water, 2 weeks	[33]
	Mice (CrL:Sk1-hrBD, weanling female hairless mice) skin	5 mg/L, sodium arsenite in drinking water, 26 weeks	[46]
	Mice (sciatic nerve denervated)	0.5 ppm, arsenic trioxide in drinking water, 4 weeks	[86]
	Mice (specific pathogen free female)	1 ppm, sodium arsenite in drinking water, 2 weeks	[68]
	Rat (Winstar, Albino male)	150 ppm (sodium arsenite), 200 ppm (sodium arsenate) in drinking water, 12 weeks	[67]
	Rat (Albino male)	25 ppm, sodium arsenite in drinking water, 8 weeks	[69]
	Rat (Albino male)	133 μg/mL (arsenic trioxide) in drinking water, 8 weeks	[71]
	Rat (Albino pregnant)	13 ppm, sodium arsenite, from gestation, lactation through full adulthood in drinking water	[72]
	Rat liver tissue in test tube	40 μM sodium arsenate uptake upto 30 min	[106]
	Rat liver mitochondria	100 μM, sodium arsenite, 5 min	[107]
	Rat liver mitochondria	25 ppm, sodium arsenite, 12 weeks	[109]
	Rat (Winstar, TR^−^)	0.5 mg/kg, 1 mmol, 0.4 mmol (MMA), 1 mmol (DMA), injection through bile duct cannulation less than 5 s	[50]
	Zebrafish	500 ppb, sodium arsenite in water, 7 days	[70]
Human patients	Arsenic biomarkers in human	1–2 mg/kg (LD_50_ of iAs), accumulated arsenic from food, water, air and soil	[29]
	Hair, nails and skin scales of arsenic-exposed patients in West Bengal, India	Arsenic patients exposed to above 50 mg/L in drinking groundwater	[44]
	Human (2–14 years old adolescents) arsenicosis patients	Upto 31.6 μg/L total inorganic arsenic exposure, upto 14 years	[66]
	Human acute promyelocytic leukaemia (APL) patients	All patients were treated with intravenous infusion of 10 mg of As_2_O_3_ (10 mL, 0.1% solution) over 2–8 h daily for 28 d and the cycle was repeated at intervals of 2 weeks until complete remission (CR) was achieved. After CR, they were maintained with several cycles of As_2_O_3_ at intervals of 2–3 months.	[87]
	Human cardiovascular disease patients	100 μg/L of arsenic above or below in meta-analysis	[95]
	Human patients (arsenic exposure)	Chronic exposure of arsenic in Bandladeshi (above 10 μg/L exposure) for 9 days	[98]
	Human patients (Diabetes)	Geographical tracing (0.1 μg/L detection limit of arsenic) in Denmark for mean follow-up for 9.7 years	[105]

## Data Availability

Data are available within the article.
